# Development of a test bench for biomechanical simulation—a preliminary study of mandibular forces

**DOI:** 10.3389/fbioe.2024.1335159

**Published:** 2024-02-26

**Authors:** Anas Ben Achour, Florian Apfeld, Günter Lauer, Christian Bräuer, Henry Leonhardt, Adrian Franke, Andreas Lipphaus, Uwe Teicher, Ulrich Witzel, Tom Alexander Schröder

**Affiliations:** ^1^ Fraunhofer Institute for Machine Tools and Forming Technology IWU, Dresden, Germany; ^2^ Biomechanics Research Group, Chair of Product Development, Faculty of Mechanical Engineering, Ruhr University Bochum, Bochum, Germany; ^3^ Department of Oral and Maxillofacial Surgery, University Hospital Carl Gustav Carus Dresden, Technische Universität Dresden, Dresden, Germany; ^4^ Department of Oral and Maxillofacial Surgery, Facial Plastic Surgery, University of Rostock, Rostock, Germany; ^5^ Else Kröner Fresenius Center for Digital Health, Technische Universität Dresden, Dresden, Germany

**Keywords:** biomechanics, bite forces, mandible, implant materials, experimental, test bench, osteosynthesis

## Abstract

**Purpose:** The aim of this study is to develop a test bench, which integrates different complexity levels and enables in that way a flexible and dynamic testing for mid and long term intervals as well as testing of maximum loads till implant failure of different osteosynthesis systems on the mandible.

**Material and Methods:** For this purpose, an analysis of the state of the art regarding existing test benches was combined with interviews of clinical experts to acquire a list of requirements. Based on these requirements a design for a modular test bench was developed. During the implementation of the test stand, functional tests were continuously carried out and improvements made. Depending on the level of complexity, the test bench can be used either as an incorporated variant or as a standalone solution. In order to verify the performance and the degree of fulfilment of the requirements of these two variants of the test bench, preliminary studies were carried out for all levels of complexity. In these preliminary studies, commercially available osteosynthesis and reconstruction plates were investigated for their biomechanical behaviour and compared with data from the literature.

**Results:** In total, fourteen test runs were performed for the different levels of complexity. Firstly, five test runs were executed to test the simplified load scenario in the incorporated variant of the test bench. High forces could be transmitted without failure of the miniplates. Secondly a quasi-static test scenario was examined using the incorporated variant with simplified load insertion. Five experiments with a number of cycles between 40,896 and 100,000 cycles were carried out. In one case the quasi-static testing resulted in a fracture of the tested reconstruction plate with a failure mode similar to the clinical observations of failure. The last four test runs were carried out using the standalone variant of the test bench simulating complex load patterns via the insertion of forces through imitated muscles. During the test runs joint forces were measured and the amplitude and vector of the resulting joint forces were calculated for both temporomandibular joints. Differences in the force transmission depending on the implant system in comparison to the zero sample could be observed.

**Conclusion:** The presented modular test bench showed to be applicable for examination of the biomechanical behavior of the mandible. It is characterized by the adjustability of the complexity regarding the load patterns and enables the subsequent integration of further sensor technologies. Follow-up studies are necessary to further qualify and optimize the test bench.

## 1 Introduction

In oral and maxillofacial surgery many different types of osteosynthesis and reconstruction plates have been developed over the last 100 years (Sauerbier et al., 2008). The challenge is to meet very different requirements in the treatment of a variety of diseases, defects or traumas. Due to the complex anatomy (Wong et al., 2010), kinematics and force transmission (Throckmorton, 2000; van Eijden, 2000; Wieja et al., 2022) of the mandible, the highest demands are placed on the plates and their attachment. Despite approaches such as patient-specific (Goodson et al., 2019) and special three-dimensional (3D) plates (de Oliveira et al., 2018), (Ben Achour et al., 2019) complications do occur, e.g., inflammations and plate exposures (van den Bergh et al., 2012), (Dean et al., 2020) or complications due to mechanical reasons like plate fractures or screw loosening (Martola et al., 2007; Probst et al., 2012; Bagheri and Bell, 2014; Seol et al., 2014). Hence they are subject to permanent further development. In order to predict the performance of these modifications of existing plate geometries respectively the new developments of plates, tests on force transmission close to reality are essential. Those results can be used to check the physical load transmission characteristics of different implants as well as for the optimization and verification of complementary Finite Element Method (FEM) simulations. For this purpose it is necessary to mimic the complex biomechanics of the mandible in its anatomical context on a laboratory scale. FEM itself has been used more recently in oral and maxillofacial surgery (Lisiak-Myszke et al., 2020). However, test benches used today by Schupp et al. (Schupp et al., (2007), Karoglan et al., (2006), Rendenhybach et al., (2017) and Zimmermann et al., (2017) do not represent these complex relationships and simplify the biomechanics by combining the various muscle forces into a single resulting force. In addition to this similarity, however, the test benches differ in their designs for force application, specimen bearing and test dynamics. Thus the comparability is limited and no precise predictions can be made about failure cases and limitations of the individual plates. In contrast to the simplified test benches, Meyer et al., (2000) chose the approach of simulating each muscle insertion by means of taped ropes for their static test bench, which can be seen as the reference test bench for static tests for complex load patterns. However, the difficulty of this test setup is the correct bonding of the ropes and the correct selection of the muscle forces. This difficulty results from the fact that these forces acting on the mandible during movement and chewing can only be estimated and are subject to a wide range as things stand today.

Besides the applied muscle forces it should also be considered to simulate the main bite forces at different points of the dentition in order to investigate the behaviour of implants during special masticatory processes. However, the maximum bite forces measured in several studies do differ in a large range mainly due to influences such as age, sex, occlusion, position of measurement and physiological and psychological conditions of test persons (Wieja et al., 2022), (Varga et al., 2011). For biomechanical testing different types of loads can be considered and used for load transmission:

• muscle forces and
• resulting bite forces.

In addition joint forces are generated whose directional vectors can be of great importance to avoid additional joint load and wear. The aim of using osteosynthesis and reconstruction plates in the mandibular region is generally to enable load transfer equivalent to the healthy mandible with preservation of the physiological stresses on the bone and the adjoining articulations over the complete period until full regeneration (Gutwald et al., 2017), (Kumar et al., 2016). However to achieve this goal flexible and dynamic testing methods for plate systems are needed that can simulate different load profiles and cover the wide range of acting forces resulting from inter-individual variations. None of the previously published test benches and methods meet these high-performance requirements for reality-based biomechanical testing of the mandible including complex load patterns and dynamics. For that reason the authors of this paper aimed to develop a test bench as an evolution of the reference test benches, which integrates different complexity levels. This is necessary in order to investigate the mid- and longterm stability and behavior of plating systems in flexible and dynamic testing as well as testing of maximum loads till implant failure. Therefore, in addition to the simplified processes for testing the maximum loads, the goal is also to enable a quasi-static test setup with consideration of the masticatory muscles as a complex case to overcome the state of the art. The main load spectrum of this complex case is implemented in a muscularly guided mastication process, which is cyclical and adaptable in its composition. Besides of those basic functions interfaces are already provided for additional sensor systems to allow expanding the range of functions of the test bench and the number of test parameters to be adapted to individual requirements. This paper describes the design of the test bench and provides the performance of each level of complexity of the test bench using results from preliminary studies that are comparable to the state of the art.

## 2 Materials and methods

### 2.1 Requirement analysis and definition of complex test benches

A flexible and dynamic test bench is supposed to realize different load scenarios in varying degree of abstraction. Therefore, the necessary requirements for the test bench are comprehensive. The analysis of specific research publications was done to use the lessons learned for the definition of minimum requirements. To achieve a high flexibility of the test bench the concept of modularity and adaptability was chosen. Based on this the following requirements for such a test bench were set.

Initially the test bench needs to be able to test various osteosynthesis systems (e.g., miniplates, 3D plates, reconstruction-plates), which are fixed on human mandible models (natural bone or artificial bone). The test bench needs to be adaptable to the size of the model. Furthermore, it is important how the forces are introduced in the mandible and how it is mounted. This aspect is dependent on the degree of abstraction. In addition, further requirements are considered in the construction (data evaluation, reproducibility and stability of the experiments, safety aspect, usability and extension capability). Based on these requirements, a test bench with two degrees of abstractions and different test scenarios was considered:

1. Simplified test scenario in which the muscles for mouth closure are represented by one resultant force, influenced by the test benches of Schupp et al. (2007), Rendenbach et al. (2017) and Zimmermann et al. (2017). This construction can be mounted on a universal stress-strain testing machine to function as a built-in system. The following requirements must be met on that variant of the test bench:
  a) Static experiments to analyze the maximal forces until failure.
  b) Quasi-static experiments to imitate simplified exposure during the chewing process.
2. Complex test scenario in which all muscles for mouth closure are realized while loading the specimen. Therefore, a new construction is necessary to function as a standalone solution. The construction is inspired by Meyer et al., (2000), who created a static test bench. With the developed test bench even quasi-static experiments are possible. Based on this the following requirements must be met to demonstrate the functionalities of the test bench and to generate approaches for new series of experiments, which overcome the state of the art:
  a) Quasi-static experiments to imitate realistic forces during the chewing process
  b) Experimental reconstruction of published force data during the chewing process, which were calculated in mathematical or simulative computations.

### 2.2 Design and components of the modular test bench

**FIGURE 1:**
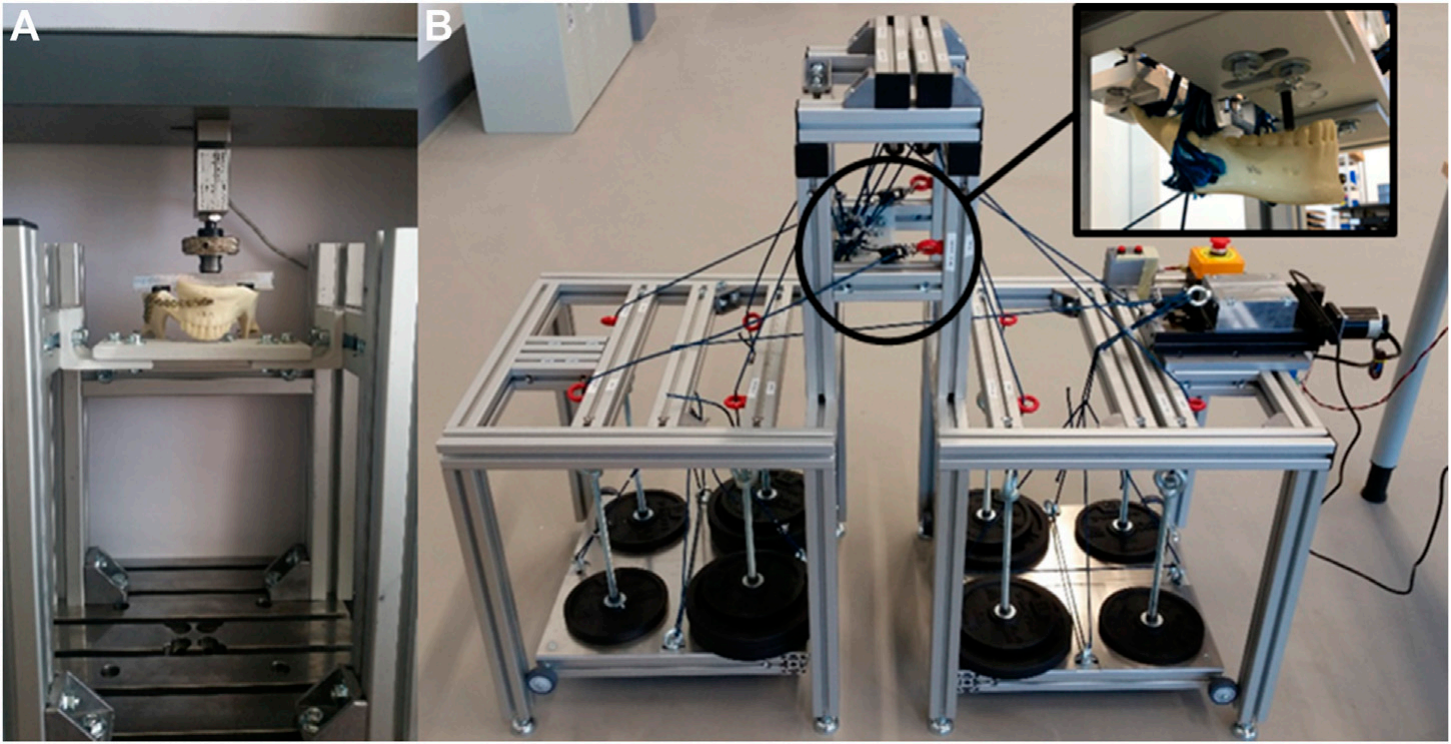
Test bench variants: **(A)** Built-in solution for simple scenarios; **(B)** Standalone solution for complex scenarios.

Both concepts are based on the same frame made of aluminium profiles, which are resistant to bending and torsion. The frame can be mounted on a universal stress-strain testing machine for the simplified concept or in the standalone test bench for the complex scenario as seen in Figure 1.

**FIGURE 2:**
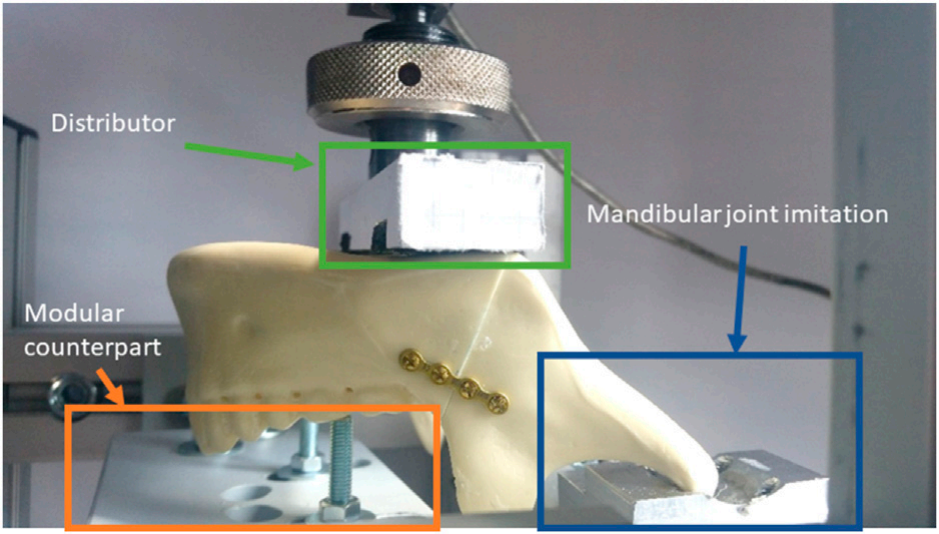
Imitation of the mandibular joint via milled aluminium part.

In both cases the mandibular joint is supported by an aluminium profile, which corresponds to the form and the function of the mandibular fossa (see Figure 2 blue box—mandibular joint imitation). The support of the resulting forces at the teeth is realized with a modular counterpart to transmit the forces via single or multiple teeth (see Figure 2 orange box—modular counterpart). Therefore a unilateral, bilateral or occlusive load scenario can be tested. Moreover, the defined force of the testing machine can be divided on the mandible via a specific distributor (see Figure 2 green box—Distributor). Therefore, it is possible to induce the force equally on both halves or split it up to 70% on the healthy side without the osteosynthesis and 30% in the affected one. This idea is based on the consideration of Schupp et al., (2007), who suggested, that the resected side will load less due to missing dental support.

The simplified test scenario was integrated in a universal stress-strain testing machine TIRAtest 2720 (TIRA GmbH, Schalkau, Germany) with an integrated control technology (EDC 222, Doli Elektronik GmbH, München, Germany) and axial force sensor (Typ Kap-S, 5kN, A.S.T. GmbH, Dresden, Germany). The generated data (force, displacement, cycle number) are transferred in the computer software TIRAtest System 4.6.0.52 (TIRA GmbH, Schalkau, Germany).

**FIGURE 3:**
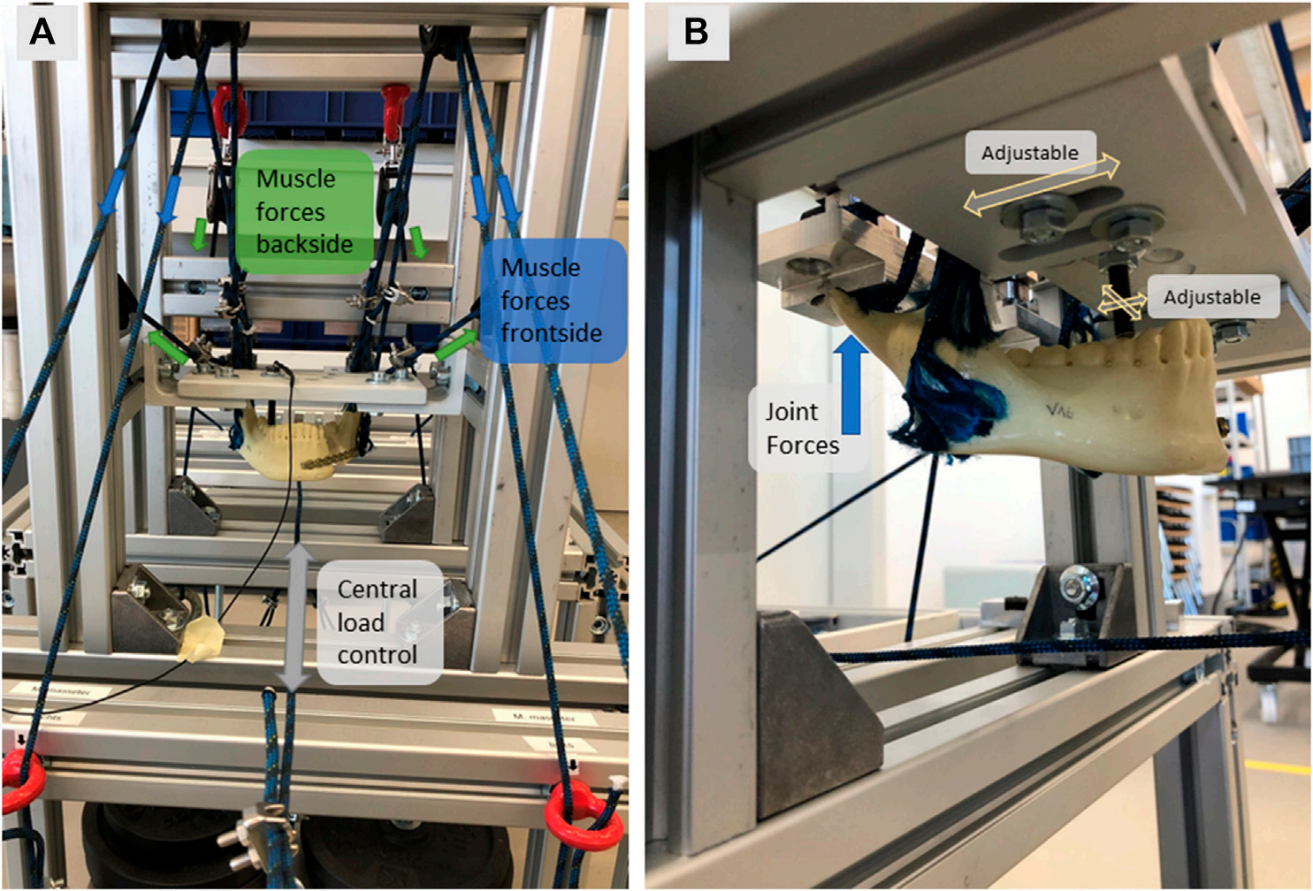
Complex load scenario with **(A)** Frontview of clamped specimen with muscle forces directing to the frontside (M.masseter, M. pt. medialis) and the backside (M.temporalis. M. pt. lateralis); **(B)** Isometric view of the clamped specimen with the adjustable position of tooth force insertion and the force transmission into the mandibular joint.

For the complex and more realistic test scenario the masticatory muscles M. temporalis, M. masseter, M. pterygoideus medialis, as well as M. pterygoideus lateralis are represented via ropes with 3 mm diameter (Regatta 2000; LIROS GmbH, Berg, Germany). The physiological vectors of each muscle force were determined by calculating a resultant angle of the individual muscle pulls of each specific muscle in the sagittal and frontal planes. The ropes are glued on the muscle insertions areas with UHU Sekundenkleber Plastic (UHU GmbH and Co. KG, Bühl/Baden, Germany), which showed the best results in tests on adhesive strength. The insertion areas are based on the illustrations of Netter (2008). With the help of guide pulleys (Sprenger GmbH, Iserlohn, Germany) the ropes are orientated in the direction of the calculated physiological muscle vectors and are connected with defined and muscle specific weights. Nevertheless, the design of the test bench enables easy adjustment of the directional vectors of the muscles if needed. The weights are laid on two movable platforms, which can be lifted vertically with one linear axis (Föhrenbach GmbH, Löffingen-Unadingen, Germany) as a central load control (see Figure 3). The motor control is realized with a stepper motor (Pythron GmbH, Gröbenzell, Germany) and the software NanoPro (Nanotec Electronic GmbH and Co. KG, Feldkirchen, Germany).

In both scenarios further force sensors can be added to measure the forces located at the mandible joint. The used sensors are piezoelectrical three axis load cells (Type 9317C, Kistler Instrumente GmbH, Winterthur, Switzerland). To measure the forces resulting on the teeth, specific strain gauges for measurements of compressive and tension forces inside of screws are tested (TB21, Hottinger Brüel & Kjaer GmbH, Darmstadt, Germany).

### 2.3 Experimental set-up of the preliminary studies to verify the test bench

After the implementation and pilot tests for optimizations the following test settings (see Table 1) were defined for the different test scenarios in order to investigate the degree of compliance of the requirements.

**TABLE 1:** Set-up of test parameters of the various test scenarios.

Scenario	Load	Distribution of force	Velocity	Stop criteria	Cycle time	Sample size n
1.1 Static	Testing machine (<2000 N)	Bilateral	5 N/s	2000 N or fracture	-	5
1.2 Quasistatic (simple)	Testing machine (400 N, 500 N, 550 N)	Unilateral	400 N/s	100.000 cycles or fracture	2.5—3.3 s	5
2. Quasistatic (complex)	Specific weights (see Table 2)	Bilateral and unilateral	Preliminary test only			

The preset parameters for each test scenario can be explained by some basic assumptions in the following way:

• **Scenario 1.1 Static tests for maximum force:**
  o Load definition: The defined load for the test is the load the testing machine realizes.
  o Load insertion: The teeth forces are located bilateral at the first molars, where the highest forces are predicted (Wieja et al., 2022; Varga et al., 2011; BAKKE et al., 1990)
  o Stop criterium: A force of 2000 N was defined as a stop criterium. In case the 2000 N were induced perfectly, every side of the mandible is strained with 1000 N. This force will be distributed between the joint and the resulted teeth force. In extensive studies, the maximum bite force is between 500 and 1000 N (Varga et al., 2011; BAKKE et al., 1990; Braun et al., 1995; van der Bilt et al., 2008; Mathur et al., 2014). In the case of osteosynthesis it certainly lower and decreases to the half of the initial strength (Tate et al., 1994).
• **Scenario 1.2 Quasistatic tests for simplified exposure:**
  oThe force is split into 30% on the mandible side with the affected and 70% on the healthy mandible side with a distributor. The teeth force is located unilaterally at the first molar of the healthy side
  oDuring the cycle of exposure the forces are alternating between a minimum force of 50 N and a maximum force of 400 N, 500 N or 550 N, depending on the experiment. This force should result in a physiological workload, which is comparable to the average during the chewing process.
  oIn contrast to other mandible test machines (Schupp et al., 2007; Rendenbach et al., 2017; Zimmermann et al., 2017), the force sensor is located differently. In this construction the resulting muscle forces are inserted via the testing machine instead of measuring the resulting force directly at the teeth. Nevertheless, the resulting teeth force can be determined with the help of the strain gauges in this test bench as well.
  oThe cycle time varies between 2.5 s (400 N) to 3.3 s (550 N), due to a constant rate of force increase
  oA maximum cycle number of 100,000 is chosen. It is assumed, that only during 2.5 min per meal, the highest exposure emerges. The remaining time is used to chew the already grinded and softened food. Due to the experimental studies mentioned in the introduction, it is expected, that the low forces (<100 N) have no significant effect on the lifetime of the osteosynthesis plates. With three meals a day and a chewing cycle time of 70 times per minute this results in 96,075 cycles for half a year. The assumptions based on the ideas of Weiskopf et al. (1981) and Verplancke et al. (2011).
• **Scenario 2. Quasistatic tests for complex exposure:**
  oThe mounting of the mandible is comparable to the quasistatic test for simple exposure (in case of unilateral exposure: force split 70:30, otherwise 50:50 with teeth force on M1).

However, in this scenario the muscle forces of four large chewing muscles on each side are represented through individual ropes with specific weights. Different load scenarios were tested (see Table 2), based on the mathematical calculations of Rues (Rues et al., 2011), (Rues et al., 2008). The loads in the bilateral load distribution are specified for each side. As can be seen, the calculated forces of Rues already differ in the results, although the experimental assumptions (bilateral unrestricted molar biting) are the same.

**TABLE 2:** Muscle forces (in N) for different load distributions to reconstruct published force data during the chewing process.

	Resulted force 1st molar	Musculus temporalis		Musculus masseter		Musculus pterygoideus medialis		Musculus pterygoideus lateralis		Joint force	
Bilateral D1	200	150		100		60		20		120	
Bilateral D2	300	230		140		100		25		300	
Unilateral D3	200	H	R	H	R	H	R	H	R	H	R
		145	105	90	80	25	30	2	5	110	140

D—Distribution set 1.3, H—Healthy side, R—Resected side.

For all experiments artificial mandibles were used (Mandible intact w/Easy Clip, serial number 8950, SYNBONE AG, Zizers, Switzerland). It is made of polyurethane and have related characteristics like human mandibles (Bredbenner and Haug, 2000). Afterwards, the fractures were cut with a bandsaw. With the help of silicone cutting guides, the fracture lines could be reproduced accurately. In the next step the osteosynthesis plates were fixed by an experienced surgeon. Different osteosynthesis and fracture scenarios were used to analyze the functionalities of the test bench:

• **Scenario 1.1:**
  oFracture along the mandibular angulus (right side), miniplate (Ti, 4 holes, 1.0 mm thickness, Anton Hipp GmbH, Fridingen a. D., Germany), 2 types of screws were tested (Ti, 2.0 × 10 mm; Ti, 2.3 × 10 mm, both Anton Hipp GmbH, Fridingen a. D., Germany)
• **Scenario 1.2:**
  oResection of a part of the mandibular corpus (left and right), reconstruction plate (Ti, 2.4 mm, Anton Hipp GmbH), 7 screws (Ti, 2.7 × 10 mm and 2.0 × 10 mm, Anton Hipp GmbH)
• **Scenario 2:**
  oFracture along the mandibular angulus (right side), miniplate (Ti, 4 holes, 1.0 mm thickness, Anton Hipp GmbH, Fridingen a. D., Germany), 2 types of screws were tested (Ti, 2.0 × 10 mm; Ti, 2.3 × 10 mm, both Anton Hipp GmbH, Fridingen a. D., Germany)Structured
  oResection of a part of the mandibular corpus (left and right), reconstruction plate (Ti, 2.4 mm, Anton Hipp GmbH), 7 screws (Ti, 2.7 × 10 mm and 2.0 × 10 mm, Anton Hipp GmbH)Structured

## 3 Results

### 3.1 Static experiments

In total five experiments of static testing were performed. The function and performance of the test bench could be confirmed. A fracture as a stop criterium occurred twice for the second and third specimen between 1,352 and 1561 N. Thereby, instead of the miniplates, the synthetic mandibular specimen broke under the mandibular condyle, where the smallest cross-sectional area of the mandible can be found and one time the side of the tooth collapsed, where the fixation of the test bench was mounted.

**FIGURE 4:**
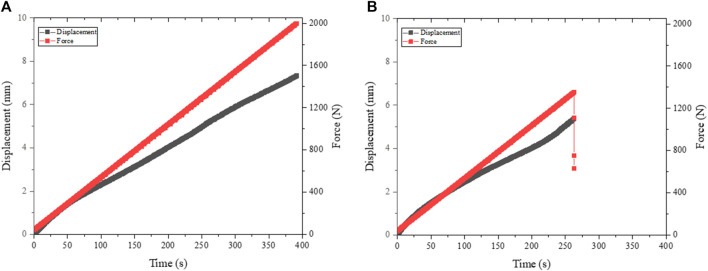
Force and displacement of static test scenarios: **(A)** Stop criterium: 2000 N and **(B)** Stop criterium: fracture.

In Figure 4 an example of the recorded force and displacement over time diagram can be seen. On the left graph a) the model could resist 2000 N. In contrast the right graph b) shows a fracture during the experiment. The universal testing machine is controlled by force (5 N/s), which explains the linear progress. The displacement progression thereby showed different shapes (some are more linear, others have a very thin s-shape). More experiments are necessary to create reasonable data to resolute this variation in displacement, to generate reliable statistics and for specific studies of osteosynthesis systems. Parallel to the machine data, it is also possible to observe the experiment with cameras to complement the knowledge of deformation. Afterwards the video and the developed crack can be analysed on the computer.

### 3.2 Quasi-static experiments for simplified exposures

**FIGURE 5:**
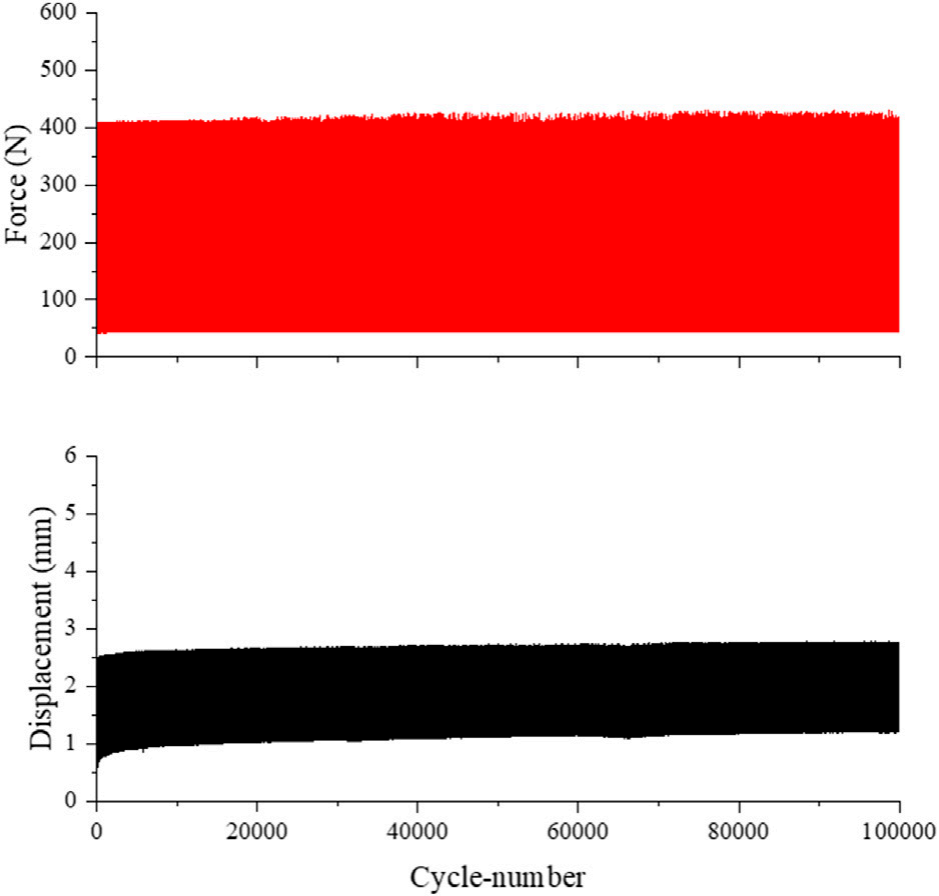
Force and displacement of the quasi-static test scenario for simplified exposure.

Five experiments were carried out. The function and performance of the test bench could be confirmed. The force-displacement curves from tests one to four are very constant and stable (see Figure 5). It can be seen that the force curve oscillates between the set maximum value of 400 N and the unloading force of approximately 50 N. The same results can be applied to the development of the displacement.

No visible damage to the reconstruction plate could be identified for the test specimens one to four. As seen in Table 3, during the preliminary tests, slight adaptions about the measuring memory, frequency and sampling rate had to be done to enable artefact free tests to achieve the preset number of 100,000 cycles. Furthermore, in following analysis of the load and displacement diagram the reliability of the measured forces was compared to the defined forces. It turns out that a regular compression tension testing machine is able to realise the quasi-static test scenario. The 90% percentile of measured peak forces has a range of only 10 N. Moreover, the effect of a smaller sampling rate on the deviation of peak forces was analysed. As can be assumed, the sampling rate of 0.15 s resulted in the best outcome and can be analysed more detailed. Even smaller sampling rates do not improve the outcome. Instead, they could even lead to errors in the measurement memory due to the amount of data.

**TABLE 3:** Test results of quasi-static tests for simplified exposure.

Specimen Nr	Cycles	Stop	Force testing machine (N)	Time (s) per cycle	Sampling rate (s)
1	66,190*	No	400	3.78	0.5
2	100,000	No	400	2.59	0.55
3	100,000	No	400	2.5	0.52
4	100,000	No	500	2.88	0.52
					0.15**
5***	40,896	Yes	550	3.29	0.5

*Test stop due to full measurement memory of the measuring program.

** From the 82,650th cycle onwards, test with higher data resolution.

*** 3 test pauses, reasons: Adjustment end criteria, adjustment due to tooth breakage.

**FIGURE 6:**
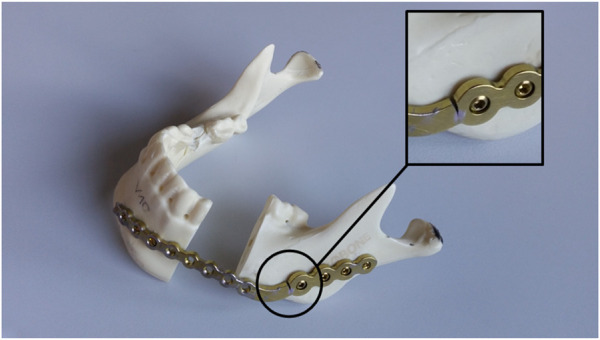
Fractured specimen and reconstruction plate of specimen 5.

Only the results of specimen number 5 are out of line, as seen in Table 3. Of all quasi-static tests, the highest compressive force of 550 N was applied to the mandibular model. Due to the adjustment of the end criteria and the control factors for a more stable test sequence, the test had to be restarted three times. Furthermore, during the test, a part of the tooth broke off, on which the tooth force was induced. As a result, the contact area for the tooth force had to be adjusted and the test bench restarted. The test finally came to a halt after 40,896 cycles due to a crack in the osteosynthesis plate (see Figure 6). A second visible crack was also found in the mandibular model itself. This crack advanced below the broken tooth. This resulted in an increased bending of the mandible model, which could be observed via larger displacement towards the end. The greater movement also influenced the reconstruction plate. In parallel, it is conceivable that the osteosynthesis plate had already weakened slightly due to fatigue and the lower stability has led to an increasing displacement. Presumably, a combination of the two effects led to a fracture of the osteosynthesis plate at the end of this test run.

### 3.3 Quasi-static experiments for complex exposures

In the preliminary study of the complex test scenario various load scenarios were tested in four test runs in total, divided into two quasi-static and two static tests. Due to the different force measurements via the three axis load cell for the joint force and a one-axis strain gauges for the teeth force, all resulting forces can be determined. This allows to have an overall comprehension of the load condition.

The measured data (Table 4) firstly show that the measured resulting forces at the temporomandibular joint are lower than the predicted calculations from the literature. Unfortunately, the tooth forces could only be determined in one case, because the force measurement with the strain gauge inside the screw did not provide reliable data over the whole time of the test runs. Only at one test run the measurement and recording of stable data was possible. In this case the described effect can be identified. However, since the tooth force could not be reliably determined, this investigation must be validated by new tests in the future.

**TABLE 4:** Results of the test runs of the complex test scenario on the standalone variant of the test bench.

Test Nr. Osteosynthesis	Distribution of muscle forces	Predicted tooth force (N)	Predicted joint force (N)	Predicted forces based on	Load modus	Tooth meshing	Measured tooth force (N)	Measured joint force (N)	Cyclenumber/cycletime
CT1 Miniplate	D2	300	300	Rues et al. (Rues et al., 2011)	bilateral	M1	?	195.4 (left)	7,486/16.3 s/cycle
								190.7 (right)	
CT2 Reconstruction plate (left side)	D3	200	150 (left)	Rues et al. (Rues et al., 2008)	unilateral	M1	?	102.4 (left)	40,280/6 s/cycle
			120 (right)					74.4 (right)	
CT3 No plate, healthy mandible	D1	200	120	Rues et al. (Rues et al., 2011)	bilateral	M1	≈220- 245	101.1 (left)	Static test only
								87.8 (right)	
CT4 Imitation of the simplified test scenario with reconstruction plate, force distributor	Resultant force of 400 N	400	30%—70% distribution	-	unilateral	M1	?	56.6 (left)	Static test only
								71.6 (right)	

CT, Complex Test 1…4, D—Distribution set (Table 2).

**FIGURE 7:**
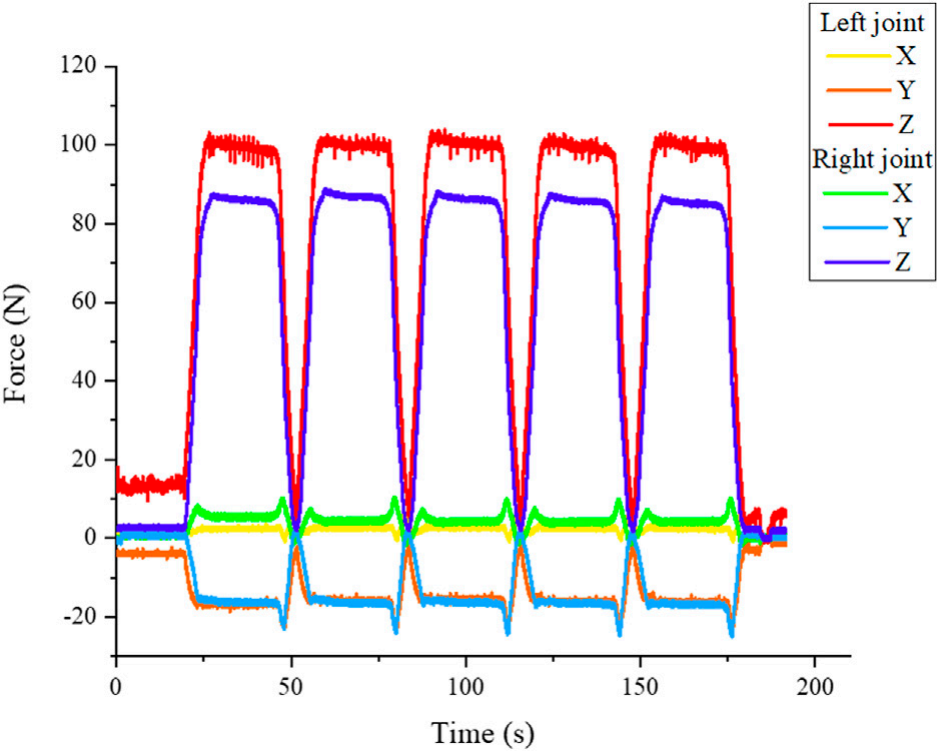
Periodic force signal of the standalone test bench shown for test run V2.

The difference between the bilateral and unilateral joint loads can also be inferred from the results. The ratio between the force of the healthy to injured side ranges from 86% (CT3) to 97% (CT1) for bilateral loading. For the unilateral loading, on the other hand, between 72% (CT2) and 79% (CT4). This tendency can be expected, since an asymmetric load consequently also entails an asymmetric force distribution. However, in this context, the result of test run CT3 stands out. Due to the bilateral force application, a value closer to 100% was expected. The result shows that an exact symmetrical force distribution can only be approximately achieved in a real test bench. An improvement is certainly needed here. However, the development of the forces over time was analysed. Therefore test run CT2 was chosen. The diagrams of the joint forces after 600 and 40,280 cycles were compared. The result was that in the local time area between a few cycles, the force progression is very similar as seen in Figure 7. In a global observation the force contribution changes a little bit. In the case of experiment 2, the resulting force decreases on both sides by 8.5 N, which is less than 10% of the maximum value.

**FIGURE 8:**
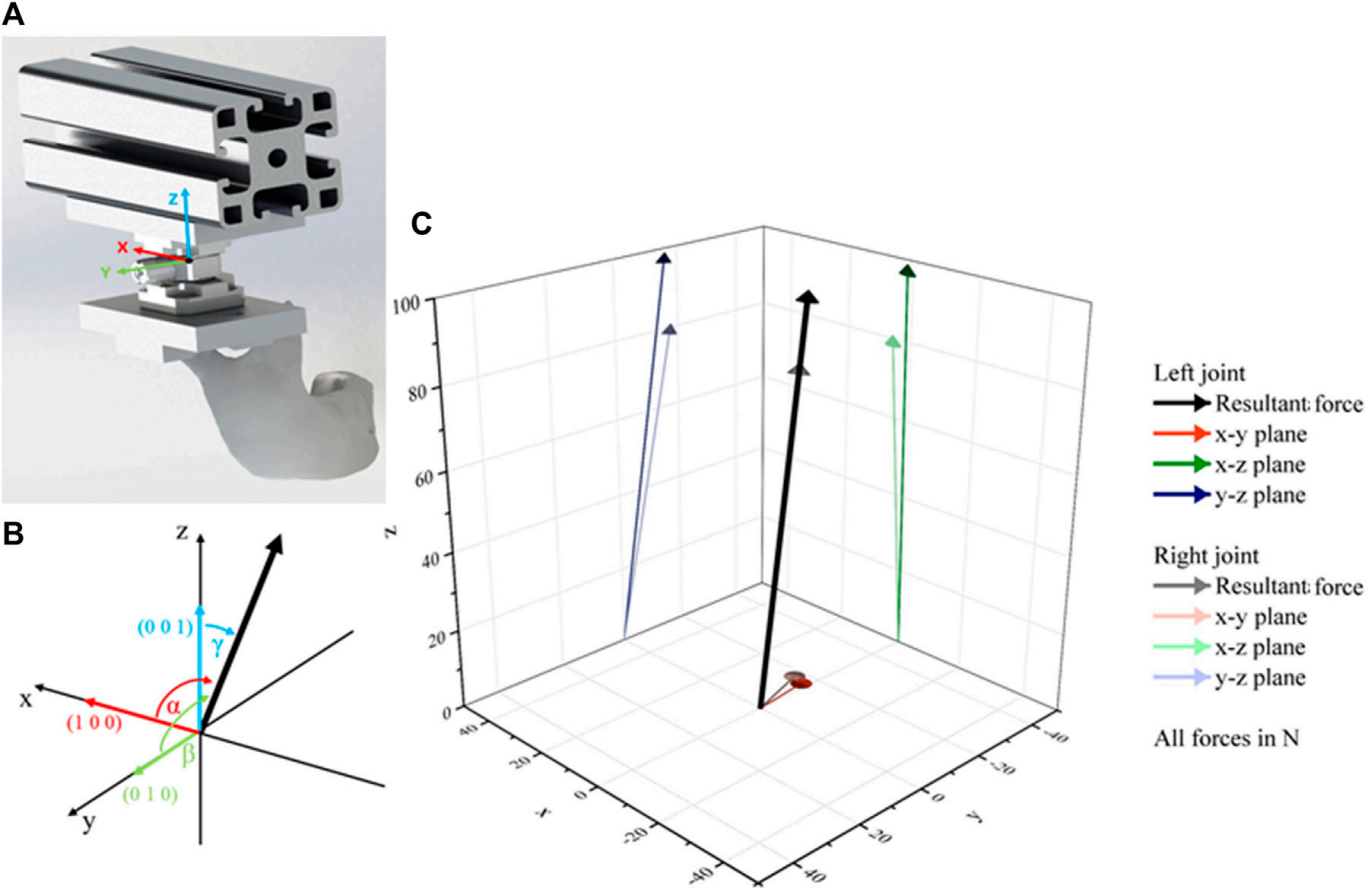
Calculation of resultant force vector with: **(A)** Location of the coordinate system, **(B)** orientation of angles and **(C)** calculated vectors of V3 at the start of measurement.

Last but not least the measured joint forces in three dimensions can be analysed during the test. Therefore, a complete cycle of loading and unloading can be visualised. As expected, the largest force is in the vertical direction z (see Figure 7 blue and red line). The course of the x- and y-directions varies significantly. The values showed that the mandible twists slightly under load (tests run CT1 and CT2) or shifts forward (test run CT3) or backward (test run CT4). A direct dependence between the movements and the type of loading or osteosynthesis plates cannot be found from the data. Reasons for that different movement can be the fixation of the system - for example, in case the mandibular condyle is not sitting perfectly in the mandibular fossa. Due to the increasing load it is pushed in the direction of the geometric centre. As a last observation a trend can be detected, that the test with bilateral loads (CT1 and CT3) affects a more symmetric load distribution compared to the unilateral load. Anyhow, it is possible to calculate based on the sensor signals the amount and the vectors of the resulting joint forces at each side of the mandible as shown in Figure 8.

Comparing the calculated angles of the resultant forces shown in Table 5 it can be seen that the orientation of the vectors does not change significantly over the duration of the test runs. The slight changes indicate again that the specimen centres itself during the test run. The almost same orientation of the left and right joint of CT3 represents the unfractured specimen and fits the expectations. In comparison to that it can be seen that the fractured and fixed specimens showed different orientations of the resulting forces on the left and right side of the mandibular. The strong difference between the test run V4 in comparison to the other fractured specimen can result from the mounting of the specimen in combination with higher implemented test forces. But there is a strong need to investigate these observations in further studies.

**TABLE 5:** Calculated angles of the vector of the resultant force.

	CT1	CT2 start	CT2 mid	CT2 end	CT3 start	CT3 end	CT4 start	CT4 end
α_left_	97	86	87	87	89	89	97	90
β_left_	96	95	94	94	99	100	71	75
γ_left_	10	6	6	5	9	10	20	15
α_right_	87	88	86	89	86	86	85	86
β_right_	75	74	75	74	102	102	61	65
γ_right_	16	16	15	16	13	11	30	25

## 4 Discussion

The estimation of the biomechanical component behaviour of osteosynthesis systems takes on a very important role for product development and optimization, especially when used for a long time and for reconstructing large defects of the mandible. The existing test benches described in the literature must evolve to enable realistic biomechanical testing as they only cover highly simplified load scenarios or static tests. However, precise knowledge of the interacting forces and torque is a key component for the realistic simulation of these complex biomechanics. Unfortunately, the published data shows that there is still no scientific consensus on the real forces acting to the mandible during different tasks. The absolute values, which are assumed for the resulting muscle force of the published experiments must also be viewed critically. Unlike the determination of masticatory forces via food experiments (Stróżyk and Bałchanowski, 2016), muscle forces cannot be measured directly, but are determined by electromyography (Gonzalez et al., 2011). Gonzalez et al. (Gonzalez et al., 2011) have shown that EMG measurements are stable in terms of the repeatability of the results. However, with EMG measurements it should be questioned whether the signals of muscles that are more difficult to access (e.g., M. pt. medialis and M. pt. lateralis) can also be reliably recorded. These aspects were not addressed in the referenced study. The exact positioning and attachment of the transducers on the skin surface could have an effect here, as well as the correct positioning of the tooth force measurement. According to Throckmorton (Throckmorton, 2000), the maximum load in the joint is already reduced by 30% if the location shifts from P2 to M1 with the same tooth force. However, based on these data and the calculated muscle forces, the resulting forces can be determined with the aid of a linear system of equations (Rues et al., 2008), which are always subject to minor deviations of the maximum values. These are sometimes accepted to solve the following system of linear equations (Rues et al., 2008). In order to approximate reality, new studies (Wieja et al., 2022), (Rues et al., 2011) now take other factors into account (e.g., the muscle feathering). In addition, there are also major variations in the ratios of the individual muscle forces within the models used. The further development of analytical calculation methods and the addition of simulations have already improved the determination of muscle forces and will continue to approximate reality in the future. Using a static resulting force, what almost all published test benches do, does not enable a change of different ratios between the single muscle forces and with that no change of the load pattern is possible. Another critical issue is that the data are mainly biased towards male patients, as they are more often considered due to the fact that they have a higher masticatory strength and the maximum values are of interest for static testing. Nevertheless, as a result of their simplification, these test benches are easy to use and can still generate valid results for certain experiments of biomechanical behavior like the maximum force transition before failure for static testing. On the other hand, the described test bench by Meyer et al. (Meyer et al., 2000), which represents a complex but static load scenario via simulating specific muscle forces, is not dynamic and cannot emulate specific load patterns over long-term periods. Moreover, this test bench is not designed to measure all kinds of forces (tooth force/teeth forces, joint forces) to acquire a complete load pattern of the mandible and to simulate deformations accordingly. In addition, due to the complex design of the test bench, reproducibility is not given as a result of the specimen holder, which is difficult to adjust.

Based on the described pros and cons, the authors decided to take the approach of a modular test bench to combine the advantages of both loading scenarios and to significantly extend their functional range. The implemented concept of the test bench showed that, in addition to the pure static tests for the investigation of maximum transferable loads, quasi-static tests could also be carried out. Furthermore, it could be shown that the dynamic force scenarios of the test routine can be adjusted within certain limits, which opens up the possibility of direct comparability with similar test benches like Schupp et al. (Schupp et al., 2007), Karoglan et al. (Karoglan et al., 2006), Rendenbach et al. (Rendenbach et al., 2017) and Zimmermann et al. (Zimmermann et al., 2017). In addition to this comparability, the test bench can be easily adapted to modified specimen geometries and new load patterns due to its sophisticated design. It is theoretically also suitable for human mandibles (natural bone) or mandibles from animal models such as minipigs. The test bench developed is outstanding in comparison with equivalent test benches due to the fact that, in addition to the resulting muscle force, the joint forces as well as the vector of these forces can also be recorded. Furthermore, an interface for inserting additional sensors for measuring the tooth force has been provided. Unfortunately the first approach of the authors to measure this force turned out to be too unstable to record reliable data. Therefore, in a future revision, the measurement principle for recording tooth forces must be adapted and validated in further experiments. The authors decided to use artificial bone specimens for the preliminary tests first of all to ensure the reproducibility by the exclusion of inter-individual variations of the specimen as they inevitably occur in natural bone. Secondly, artificial bone was used to compare the results of preliminary testing with published results of other experiments. For that reason it can be proven that the results of the static test scenario showed a similar outcome in comparison with Zimmermann et al. (Zimmermann et al., 2017). Only the absolute values of the calculated stiffness differ between the two test benches as a result of the different manufacturers of the osteosynthesis systems and the resulting component deviations with slightly different bearings. All mini-plates tested were able to transmit the applied forces without component failure.

Comparing the quasi-static results for simplified load patterns with published results of Schupp et al. (Schupp et al., 2007) and Karoglan et al. (Karoglan et al., 2006) it can be seen that the deformation of the mini-plate fixed specimen and the mode of failure of the reconstruction plates of this preliminary study are similar to the published ones. In order to achieve exact comparability between the test benches described in literature and this one, it would be necessary to be able to set the exact tooth force for the author’s test bench. This was unfortunately not possible due to the unstable sensor system of the screw-specimen contact, so that the tooth forces had to be estimated on the basis of the system of equations from Rues et al. (Rues et al., 2008) and the directly measured joint forces. It has been shown that using a resulting force of 400 N produces approximately 150 N of tooth force and asymmetric joint forces. This resulting force was too low to cause significant failure events in the quasi-static test for all specimens. By increasing the resulting force to 550 N it could be shown that the quasi-static testing results in higher tooth forces and with that in a typical breakage of the reconstruction plates as described in Schupp et al. (Schupp et al., 2007) and Rendenbach et al. (Rendenbach et al., 2017). The reconstruction plates break mainly in the region next to the first screw hole of the tension side of the plate, where the cross section is the smallest.

The measured data of the quasi-static modus for complex load pattern firstly showed that the measured resulting forces at the temporomandibular joint are lower than the predicted calculations from the literature. The reasons for this can be manifold. Even slight deviations at the muscle insertion surfaces could lead to changes. Rues et al. (Rues et al., 2011), (Rues et al., 2008) assume a defined start and end point for the calculations, whereas an insertion surface is used in the test model. Furthermore, it is possible that a slight deviation of the muscle angle may impact on the result. The same issue can be applied to the exact tooth insertion point on the first molar. The sum of the small deviations can ultimately lead to different results. Another important reference variable in this context is the tooth force. It is possible that in the present test bench a larger force is transmitted through the teeth, which would compensate for the lower joint forces. When comparing the force distribution of the complex scenario via measuring the joint forces it could be seen that an exact symmetrical force distribution for the unfractured and mini-plate fixed specimen can only be achieved approximately in a real test bench. In fact, these minor deviations also occurred in the simplified test rig of Karoglan et al. (Karoglan et al., 2006). Deviation from the symmetric load distribution can be caused by different factors, e.g., slight changes in the clamping of the specimen or the position of the counterpart to insert tooth forces or even material defects of the specimen. There was also a deviations between the expected and measured values of the joint forces (30%–70%) when using the force distributor while testing the reconstructed specimen. Those deviations can be caused due to the fine positioning of the distributor or counterpart. Another point is that in the case of testing a reconstruction plate with unilateral loading, the affected side absorbs a greater joint force (CT2, CT4). Since along the resected side, no force can be transferred via the teeth but only via the temporomandibular joint, the result seems quite plausible. Another very interesting point is that by imitating the simplified load pattern (CT4) on the complex standalone version of the test bench the orientation and amount of the joint forces differ notably in comparison to the complex load pattern (CT2). This clearly indicates that the force transmission through the mandible and the bearing force on the tooth-specimen-contact are dependent on the complexity of the test bench. Simplified test benches can therefore lead to a biased result with regard to the biomechanical suitability of implants in this case. This bias can be avoided by using load patterns that are close to reality. The test bench for complex load patterns presented in this paper produces load patterns which are close to reality. It can be used already for quasi-static long-term tests of implant systems in order to investigate their biomechanical behavior and represents the basic solution for upcoming evolvements.

## 5 Conclusion

In conclusion the presented modular test bench showed to be applicable for the examination of the biomechanics of the mandible. Based on the results of the preliminary studies it can be observed, that the published of established test benches could be viable reproduced. Furthermore, it could be proven, that using a complex load pattern via reality-based muscle forces and the measurement of all forces in quasi-static mastication processes is considered reasonable. The presented standalone solution of the test bench significantly exceeds the state of the art due to its quasi-static test execution for the simulation of long time periods as well as the flexible adjustability of muscle forces and direction vectors and the inclusion of different specimen types and geometries. For this reason, the complex experimental setup presented in this paper should be further developed for future investigations. For this purpose, the test bench must be extended to include a robust and accurate solution for measuring the tooth force as well as an integration of control loops for adaptive adjustment of the muscle forces during long-term test runs to simulate different diets during the regeneration phase of the patient.

## Data Availability

The original contributions presented in the study are included in the article/Supplementary material, further inquiries can be directed to the corresponding author.
